# Current challenges and unmet medical needs in myelodysplastic syndromes

**DOI:** 10.1038/s41375-021-01265-7

**Published:** 2021-05-28

**Authors:** Uwe Platzbecker, Anne Sophie Kubasch, Collin Homer-Bouthiette, Thomas Prebet

**Affiliations:** 1 Department of Hematology, Cellular Therapy and Hemostaseology, Leipzig University Hospital, Leipzig, Germany; 2 German MDS Study Group (D-MDS), Leipzig, Germany; 3 The European Myelodysplastic Syndromes Cooperative Group (EMSCO), Leipzig, Germany; 4 Yale School of Medicine, New Haven, CT USA

**Keywords:** Haematological diseases, Clinical genetics

## Abstract

Myelodysplastic syndromes (MDS) represent a heterogeneous group of myeloid neoplasms that are characterized by ineffective hematopoiesis, variable cytopenias, and a risk of progression to acute myeloid leukemia. Most patients with MDS are affected by anemia and anemia-related symptoms, which negatively impact their quality of life. While many patients with MDS have lower-risk disease and are managed by existing treatments, there currently is no clear standard of care for many patients. For patients with higher-risk disease, the treatment priority is changing the natural history of the disease by delaying disease progression to acute myeloid leukemia and improving overall survival. However, existing treatments for MDS are generally not curative and many patients experience relapse or resistance to first-line treatment. Thus, there remains an unmet need for new, more effective but tolerable strategies to manage MDS. Recent advances in molecular diagnostics have improved our understanding of the pathogenesis of MDS, and it is becoming clear that the diverse nature of genetic abnormalities that drive MDS demands a complex and personalized treatment approach. This review will discuss some of the challenges related to the current MDS treatment landscape, as well as new approaches currently in development.

## Introduction

Myelodysplastic syndromes (MDS) represent a heterogeneous group of myeloid neoplasms that are characterized by inefficient hematopoiesis, variable cytopenias, and a risk of progression to acute myeloid leukemia (AML). The incidence rate of MDS in the general population is 4.5 per 100,000 people per year; incidence is higher in males than females (6.2 vs. 3.3 per 100,000 people per year) and substantially rises with age [1]. The incidence rate is low in individuals aged <40 years, at ~0.1 per 100,000 people per year, rising to 26.9 per 100,000 people per year in those aged 70–79 years and further to 55.4 per 100,000 people per year among those aged ≥80 years [1, 2]. Other risk factors for MDS include prior treatment with certain chemotherapy drugs or radiation therapy (known as therapy-related MDS and classified under therapy-related myeloid neoplasms) and environmental exposure, such as long-term workplace exposure to benzene and other chemicals [2–4]. Although familial forms of MDS are rare, genetic predisposition is increasingly recognized; bone marrow failure syndromes (e.g., Fanconi anemia, dyskeratosis congenita, Diamond-Blackfan anemia, and Shwachman-Diamond syndrome in children) have been shown to significantly increase the risk of MDS [5]. A better understanding of genes that may predispose MDS, the development of a comprehensive list of these genes, and more consistent testing for these genes may help to improve the management of patients with MDS [6].

Most patients with MDS are affected by anemia and anemia-related symptoms, which negatively impact health-related quality of life (QoL) [7]. For example, in an online survey of patients with MDS, 82% of respondents reported anemia at baseline, with significantly worse patient-reported outcomes in those with higher-risk than lower-risk MDS [8]. Further, anemia and transfusion dependence are associated with shorter survival in patients with MDS [9]. In an observational study of patients with lower-risk MDS, the most important independent predictor of health-related QoL was hemoglobin level, which is associated with fatigue levels [7]. Two additional surveys of patients with MDS provide further support that hemoglobin levels and fatigue appear to have the greatest impact on patient-reported outcomes [10, 11]. Results from these studies reinforce the serious impact of anemia/MDS on patient-reported outcomes, including the burden of blood transfusions [8, 10, 11]. While many patients with MDS have lower-risk disease and are managed by existing treatments or a watch-and-wait strategy, there is no standard of care for the majority of these patients; many are not candidates for approved treatments or experience relapse after first-line treatment and require further therapy [12]. Thus, there remain many challenges and unmet needs for patients with MDS. This review will discuss some of the challenges related to the current MDS treatment landscape, as well as new approaches in development for the treatment of MDS.

## Pathophysiology and diagnosis of MDS

### MDS pathophysiology

The clinical presentation of MDS is usually nonspecific. Patients present with signs and symptoms associated with cytopenias, such as fatigue resulting from anemia, infections due to neutropenia, and/or bleeding and bruising from thrombocytopenia and thrombocytopathy, which trigger a diagnostic workup for MDS [13, 14].

Recent advances in molecular diagnostics have improved our understanding of the pathogenesis of MDS through the identification of cytogenetic abnormalities and gene mutations. Cytogenetic abnormalities occur in approximately half of patients with MDS [15], with the most common sole abnormality being deletion in 5q (del[5q]) [15, 16]. However, ≥1 DNA mutation may be found in 70–80% of the patients [17]. Multiple gene mutations have been found in patients with MDS and involve genes responsible for epigenetic regulation (*TET2*, *ASXL1*, *EZH2*, *DNMT3A*, and *IDH1/2*), RNA splicing (*SF3B1*, *SRSF2*, *U2AF1*, and *ZRSR2*), DNA damage response (*TP53*), transcriptional regulation (*RUNX1*, *BCOR*, and *ETV6*), signal transduction (*CBL*, *NRAS*, and *JAK2*), and the cohesion complex (*STAG2*) [16, 18]. Genetic causes of MDS appear to be diverse, as no single mutated gene is found in more than a third of MDS patients [19, 20]. Several gene mutations, such as *TP53*, *EZH2*, *ETV6*, *RUNX1*, and *ASXL1*, are associated with adverse clinical features and may also hold independent prognostic value [19, 21–23]. Further, an assessment of the impact of the allelic state of *TP53* on clinical outcomes in MDS patients suggested bi-allelic, but not mono-allelic, *TP53* mutations are associated with high-risk disease, poor survival, and rapid transformation to leukemia [24]. Mutation profiling can thus help to refine risk categorization and identify appropriate therapies, including targeted therapies for patients with certain mutations [20]. Large-scale mutation profiling can also refine prognosis for both low- and high-risk MDS [17, 21, 25, 26]. It is important to note that a patient’s mutation profile typically changes from diagnosis to relapse after chemotherapy or hematopoietic cell transplantation (HCT), with both gains and losses of mutations commonly identified [27], highlighting the importance of re-evaluating the mutation profile after relapse and prior to initiating subsequent lines of therapy. Of note, the spectrum of mutations seen in MDS overlaps significantly with other conditions related to MDS, such as clonal hematopoiesis of indeterminate potential, clonal cytopenias of undetermined significance, and, on the other end of the spectrum, AML [28].

MDS is associated with immune dysregulation and an increased release of inflammatory cytokines, including tumor-necrosis factor alpha, interferon-gamma, transforming growth factor-ß, and interleukins (e.g., IL-6, IL-10), which are expressed by mesenchymal stromal cells, hematopoietic cells, and T cells in the bone marrow microenvironment [29, 30]. Mesenchymal stromal cells are critical for the regulation of hematopoietic stem and progenitor cells, aiding in the reinforcement of clonal dominance of MDS cells, and additionally suppress T-cell proliferation and activation [30]. MDS is also associated with an increase in myeloid-derived suppressor cells (MDSCs), which mediate a pro-inflammatory response and potently suppress T-cell function [29, 30]. MDSCs are activated through the binding of S100A8 and S100A9 to toll-like receptor (TLR)-4 and CD33 and contribute to innate immune activation. The expression of the secretion of S100A8 and S100A9 by activated MDSCs results in autocrine and paracrine stimulation, with downstream activation of the NLRP3 pattern recognition receptor and subsequent inflammasome assembly and pyroptosis. In MDS patients who have a del(5q) phenotype, the induction of S100A8 and S100A9 also leads to a p53-dependent defect in erythroblast differentiation [29, 30]. In addition to the pro-inflammatory state and innate immune activation seen in MDS, adaptive immune cell function is also impaired. Regulatory T-cell counts are decreased in the peripheral blood of patients with low-risk MDS but undergo expansion in patients with higher-risk MDS, indicating progressive immunosuppression with advancing disease. Cytotoxic CD8^+^ T-cell and natural killer cell counts are increased in MDS patients compared to healthy individuals [30].

### MDS diagnosis and classification

Although abnormal findings on routine blood testing often lead to suspicion of MDS, diagnosis is confirmed via bone marrow aspiration (cellular morphology and percentage of blasts), bone marrow biopsy (cellularity and architecture), and cytogenetic or molecular genetic analysis [31]. An analysis of bone marrow cytogenetics is also required to help calculate a prognosis score and may be helpful in treatment selection for certain genetic mutations [31]. Although most patients carry ≥1 somatic genetic mutation, these mutations are not part of the current diagnostic criteria for MDS, with the exception of *SF3B1* mutations [31]. The 2016 World Health Organization (WHO) guidelines identify seven morphologic subtypes of adult MDS, which are summarized in Table 1 [19, 32].

**Table 1:** 2016 WHO classification of MDS [19, 32].

	Dysplastic lineages	Cytopenias^a^	Blasts in blood	Blasts in bone marrow
MDS with single-lineage dysplasia (MDS-SLD)	1	1 or 2	<1%	<5%
MDS with multilineage dysplasia (MDS-MLD)	2 or 3	1–3	<1%	<5%
MDS with ring sideroblasts (MDS-RS)^b^				
MDS-RS-SLD	1	1 or 2	<1%	<5%
MDS-RS-MLD	2 or 3	1–3	<1%	<5%
MDS with excess blasts (MDS-EB)				
MDS-EB1	0–3	1–3	2–4%	5–9%
MDS-EB2	0–3	1–3	5–19%	10–19%
MDS with isolated del(5q)	1–3	1–2	<1%	<5%
MDS, unclassifiable (MDS-U)^c^	0–3	1–3	≤1%	<5%

*MDS* myelodysplastic syndromes, *PB* peripheral blood, *WHO* World Health Organization.

^a^Cytopenias defined as hemoglobin <10 g/dl, platelet count <100 × 10^9^/l, and absolute neutrophil count <1.8 × 10^9^/l. In rare cases, MDS may present with mild anemia or thrombocytopenia above these levels. PB monocytes must be <1 × 10^9^/l.

^b^Patients have ≥15% ring sideroblasts in marrow or ≥5% with *SF3B1* mutation.

^c^MDS-U includes patients with 1% blood blasts, SLD and pancytopenia, or MDS-U based on defining cytogenetic abnormality.

Prognostic scoring systems are essential for initial patient stratification and subsequent treatment decision. The International Prognostic Scoring System (IPSS) was developed in 1997 and includes percentage of blasts, number of cytopenias, and presence of cytogenetic abnormalities to classify patients into 4 risk categories (low, intermediate-1 [INT-1], intermediate-2 [INT-2], and high) [33]. The WHO classification-based Prognostic Scoring System incorporates the WHO morphologic categories, the IPSS cytogenetic categories, and the presence or absence of severe anemia (hemoglobin < 9 g/dl for males and <8 g/dl for females) to classify patients into 5 risk categories (very low, low, intermediate, high, and very high) [34, 35]. A revised version of the IPSS (IPSS-R) is currently the accepted standard scoring system and includes refined cytogenetic risk classification, improved stratification by bone marrow blast counts, and more clearly defined degrees of cytopenias to classify patients into 5 risk categories (very low, low, intermediate, high, and very high) [12, 36]. The incorporation of molecular data into the IPSS-R is currently being evaluated. The Lower-risk Prognostic Scoring System was developed to better identify patients with lower-risk MDS (including low- and intermediate-risk patients) who may have poor prognosis and may benefit from early intervention [37]. Unfavorable cytogenetics, older age (≥60 years), decreased hemoglobin (<10 g/dl), decreased platelet count (<50 × 10^9^/l), and higher percentage of bone marrow blasts (≥4%) were associated with worse risk, with age and low platelet counts being the most important factors [37]. The results from this model show that patients with lower-risk MDS and poor prognosis may benefit from early intervention [37].

Despite these prognostic risk classifications, therapeutic options are limited for the majority of patients with MDS, and, in particular, patients with intermediate risk represent a heterogeneous group of patients who may have favorable or unfavorable disease courses [12]. Risk stratification can be improved by consideration of gene mutations, comorbidities, and frailty index [19, 21, 38]. Age ≥66 years, peripheral blood blasts ≥2%, and history of red blood cell (RBC) transfusion have also been identified as stratification factors for patients with intermediate-risk MDS [39].

## Therapeutic approaches for MDS

Treatment goals for patients with MDS are two-fold: improve peripheral blood values (i.e., increase hemoglobin levels and reduce bleeding and infections) and change the natural progression of the disease [40]. The choice of therapy for newly diagnosed and relapsed/refractory MDS depends on the individual patient’s risk classification, fitness (including comorbidities), goals and preferences, caregiver and social support, and suitability for HCT. The National Comprehensive Cancer Network (NCCN) MDS Panel recommends stratifying patients with clinically significant cytopenia(s) into lower- and higher-risk groups [19]. Lower-risk patients include those with IPSS low or INT-1 classification; IPSS-R very low, low, or intermediate classification up to 3.5 points; or WHO classification-based Prognostic Scoring System very low, low, or intermediate. Higher-risk patients are those classified as IPSS INT-2 or high; IPSS-R intermediate (>3.5 points), high, or very high; or WHO classification-based Prognostic Scoring System high or very high [19]. Patients with IPSS-R intermediate MDS can be managed as low- or high-risk MDS based on other prognostic factors (e.g., age, performance status, mutations, serum ferritin levels, and serum lactate dehydrogenase levels) [19].

**Fig. 1:**
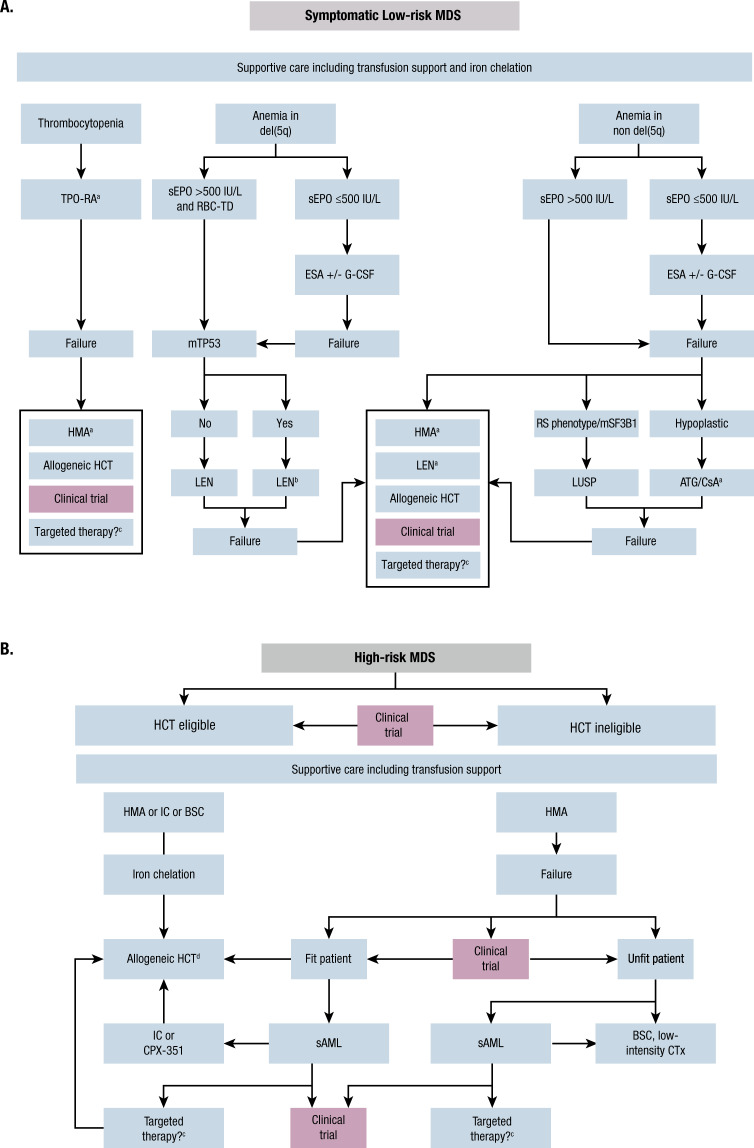
Guideline-recommended treatment options for MDS [41]. **A** Symptomatic low-risk MDS. **B** High-risk MDS. ^a^Not presently approved. ^b^Intensified disease surveillance. ^c^These could be IDH or FLT3 inhibitors (not presently approved). ^d^Consider posttransplant disease surveillance strategies. ATG antithymocyte globulin, BSC best supportive care, CsA cyclosporine, CTx chemotherapy, ESA erythropoiesis-stimulating agent, G-CSF granulocyte colony-stimulating factor, HCT hematopoietic cell transplantation, HMA hypomethylating agent, IC induction chemotherapy, LEN lenalidomide, LUSP luspatercept, MDS myelodysplastic syndromes, RBC-TD red blood cell transfusion dependence, RS ring sideroblast, sAML secondary acute myeloid leukemia, sEPO serum erythropoietin, TPO-RA thrombopoietin receptor agonist. This research was originally published in *Blood*; both figures have been adapted from the original publication. U Platzbecker. Treatment of MDS. *Blood*. 2019;133:1096-1107. © the American Society of Hematology.

The main treatment goals for lower-risk MDS are hematologic improvement to prevent complications (e.g., bleeding and severe infections), decreased transfusion burden, and improved QoL; accordingly, endpoints in trials for lower-risk MDS should therefore also focus on QoL [12, 19, 41]. For patients with higher-risk MDS, the treatment priority is changing the natural history of the disease by delaying disease progression, improving overall survival, and proceeding to HCT, if possible, to potentially achieve a cure [12, 19, 41]. Before initiating treatment for high-risk MDS, patients should be evaluated for candidacy for HCT, including age and comorbidities (Fig. 1) [41]. All patients, regardless of risk, should receive supportive care measures as part of the MDS therapeutic algorithm, comprising observation, clinical monitoring, psychosocial support, and QoL assessment [19]. Supportive care includes RBC transfusions for symptomatic anemia or platelet transfusions for bleeding events, antibiotics for bacterial infections, and iron chelation for iron overload (for patients with low-risk MDS). For high-risk MDS, iron chelation is recommended preferentially for those responding to hypomethylating agent (HMA)-based therapy or being scheduled for HCT (Fig. 1) [19, 41]. Neutropenia remains an unmet medical need for many patients with MDS and can be associated with recurrent and/or serious infection [19, 42]. In low-risk MDS, granulocyte colony-stimulating factor is recommended for patients with life-threatening infections. In high-risk MDS, agents with the ability to change the natural course of the disease (i.e., HMAs) should be preferred.

### Summary of current treatment options

A summary of current treatment options for patients with lower-risk and higher-risk MDS is provided in Fig. 1.

#### Erythropoiesis-stimulating and maturing agents (ESAs and EMAs)

ESAs, such as recombinant erythropoietin or darbepoetin, are a standard first-line therapy for anemia in patients with lower-risk MDS [41, 43, 44]. These agents are recommended by the NCCN and European LeukemiaNet (ELN) for the management of symptomatic anemia in lower-risk MDS patients with a target hemoglobin range of 10–12 g/dl [19, 44]. The level of serum erythropoietin (sEPO) is a strong predictor of clinical response to ESAs; patients with lower-risk MDS with a sEPO level of <100 U/l have a response rate of >70%, whereas, for those patients with a sEPO level of >500 U/l, the response rate is <10% [45]. A prospective, randomized, phase 3 study compared the outcomes of patients treated with erythropoietin with or without granulocyte colony-stimulating factor plus supportive care vs. supportive care alone in 118 anemic patients with lower-risk MDS. The response rates in the erythropoietin vs. supportive care alone arms were 36% vs. 10%, respectively, at the initial treatment step; response rate in the erythropoietin arm increased to 47% following step 4 therapy [46].

Luspatercept and sotatercept (which are not currently approved for the treatment of MDS) are specific activin receptor IgG-Fc fusion proteins that act as ligand traps to neutralize negative regulators of late-stage erythropoiesis that have shown efficacy in phase 2 and phase 3 clinical studies [47–49]. In an open-label, phase 2 study in patients with lower-risk MDS, luspatercept was found to be particularly effective in the treatment of anemia in patients who had ≥15% ring sideroblasts, an *SF3B1* mutation, or both [50]. The COMMANDS study is currently evaluating a head-to-head comparison of luspatercept in patients with MDS with or without ring sideroblasts (ClinicalTrials.gov Identifier: NCT03682536). Luspatercept was recently approved by the US Food and Drug Administration (FDA) for the treatment of anemia failing an ESA and requiring ≥2 RBC units over 8 weeks in adult patients with very low- to intermediate-risk MDS with ring sideroblasts or with myelodysplastic/myeloproliferative neoplasm with ring sideroblasts and thrombocytosis based on the results of the phase 3 MEDALIST study [48]. Luspatercept was also approved by the European Medicines Agency for the treatment of anemia that requires regular blood transfusions in adult patients with MDS [51]. Clinical results with sotatercept are comparable to those with luspatercept [47] despite differences in ligand affinities.

#### Low-intensity therapy

##### Hypomethylating agents

Low-intensity therapies include HMAs or biologic response modifiers. HMAs (azacitidine, intravenous decitabine, and oral decitabine) are approved by the FDA for the treatment of MDS; azacitidine is approved for the treatment of various MDS subtypes (refractory anemia or refractory anemia with ring sideroblasts, excess blasts, or excess blasts in transformation, and chronic myelomonocytic leukemia; IPSS risk category not mentioned) [52], and decitabine is approved for IPSS INT-1- or higher-risk patients (all MDS subtypes) in the United States [53], and for patients with newly diagnosed *de novo* or secondary AML who are not candidates for standard induction chemotherapy in Europe [54]. HMAs are a first-line treatment option for higher-risk MDS [12, 41, 43] and are recommended by the NCCN for patients with IPSS INT-2- or high-risk disease or IPSS-R intermediate-, high-, or very high-risk disease with any of the following criteria: the patient is not a candidate for high-intensity therapy, potential candidate for allogeneic HCT but for whom delay in receipt of that procedure is anticipated, or not expected to respond to (or relapsed after) ESAs or immunosuppressive therapy [19]. The ELN recommends the use of azacitidine in patients with IPSS INT-2- or high-risk disease who are not eligible for AML-like chemotherapy and/or allogeneic HCT and in fit patients with IPSS INT-2- or high-risk MDS and poor-risk cytogenetics who lack a donor; it can also be offered to fit patients without poor-risk cytogenetics who lack a donor as an alternative to induction chemotherapy [44]. Possible predictors of response to HMA treatment have been identified and include gene mutations such as *TET2* in the absence of *ASXL1* (presence is associated with better response), although data are conflicting; clinical parameters such as older age, male sex, and Eastern Cooperative Oncology Group performance status >1; high transfusion burden; and poor cytogenetics (associated with worse outcomes) [55].

##### Immunosuppressive therapy

The NCCN recommends immunosuppressive therapy with antithymocyte globulin (ATG) with or without cyclosporine A in patients aged <60 years with ≤5% blasts or in those with hypocellular marrows, paroxysmal nocturnal hemoglobinuria clone positivity, or *STAT-3* mutated cytotoxic T-cell clones [19]. The ELN states that ATG plus 6 months of oral cyclosporine A should be considered in patients aged <60 years with <5% bone marrow blasts, normal cytogenetics, and transfusion dependency who are not candidates for hematopoietic growth factors; ATG is highly recommended in the presence of a hypoplastic bone marrow [44]. Patients more likely to respond to ATG are those with MDS with single-lineage dysplasia with absence of ring sideroblasts, hypoplastic marrow, DR15 HLA type, age <60 years, female sex, trisomy 8, and short duration of transfusion dependence [41]. In a large, international, retrospective cohort study of 207 patients with low-risk MDS, 76% of patients received ATG-based combinations, with the most common being ATG plus prednisone (43%) [56]. The overall response rate was 49%, including 11% who achieved a complete response; 30% achieved RBC transfusion independence. The median duration of transfusion independence was 19.9 months [56].

##### Immunomodulatory drugs

Lenalidomide is approved by the European Medicines Agency for the treatment of transfusion-dependent anemia in IPSS low- or INT-1-risk MDS patients with del(5q) [57] and the FDA for the treatment of transfusion-dependent anemia in IPSS low- or INT-1-risk MDS patients with del(5q) with or without additional cytogenetic abnormalities [58]. The NCCN Guidelines Panel recommends lenalidomide for patients with lower-risk MDS with del(5q) chromosomal abnormalities alone or with other cytogenetic abnormality (except those involving chromosome 7) and symptomatic anemia or in patients with symptomatically anemic non-del(5q) MDS with anemia that did not respond to initial therapy [19]. The ELN recommends lenalidomide in patients with del(5q) without additional chromosomal abnormalities or excess blasts with a low or INT-1 IPSS score and transfusion-dependent anemia who are not candidates for or have failed treatment with hematopoietic growth factors [44]. Results from a retrospective cohort study suggest the presence of *TP53* mutations may predict disease progression in MDS patients with lower-risk del(5q) treated with lenalidomide [59]. In a randomized, double-blind, phase 3 study in patients with lower-risk non-del(5q) MDS, RBC transfusion independence ≥8 weeks was achieved in 27% vs. 2.5% of patients treated with lenalidomide vs. placebo, respectively, with a median duration of RBC transfusion independence of 30.9 weeks with lenalidomide [60]. The most frequently reported adverse events were neutropenia and thrombocytopenia [60].

#### High-intensity therapy

High-intensity therapy includes intensive induction chemotherapy or HCT. Because of the greater risks of regimen-related morbidity and mortality with these regimens, these treatments are recommended only in the context of clinical trials [19].

##### Intensive induction chemotherapy

The NCCN guidelines state that intensive induction chemotherapy should be considered for patients who are eligible for intensive therapy but lack a suitable donor or for patients who need reductions in bone marrow blast counts [19]. Although responses are not as high and durable as those observed for AML, treatment could be beneficial. Intensive chemotherapy is recommended for patients with ≥10% bone marrow blasts who are candidates for allogeneic HCT within a clinical trial or prospective registry [44, 61]. The ELN guidance recommends that induction chemotherapy should be considered for fit patients without a suitable donor who are ≤65 to 70 years of age and have ≥10% bone marrow blasts without adverse cytogenetic characteristics [44]. A retrospective analysis of 299 patients with high-risk MDS or secondary AML from the Duesseldorf MDS registry showed that conventional intensive chemotherapy did not lead to a significant improvement in median overall survival vs. non-intensive therapy (12.7 vs. 7 months; log rank *P* = 0.381) [62].

##### Hematopoietic cell transplantation

Important variables to consider when determining patient eligibility for HCT are IPSS, IPSS-R, age, comorbid conditions, psychosocial status, patient preference, and availability of a suitable donor and caregiver [19, 44, 61, 63]. The HCT-comorbidity index is a tool for determining pre-HCT comorbidities that can be used for predicting outcomes and stratifying patients for HCT [64, 65]; a composite HCT-comorbidity index/age index has also been developed that takes into account both the burden of comorbidities and increasing age when determining risk assessment [65]. Candidates for allogeneic HCT are fit, aged ≤65 to 70 years, and IPSS INT-2 or high-risk, or IPSS INT-1 risk with excess blasts or poor-risk cytogenetics [44, 61]. The ELN and NCCN both state that peripheral blood stem cells are the preferred source for allogeneic HCT from an HLA-matched donor in patients with MDS [19, 44]. Either an HLA-matched sibling or an HLA-matched unrelated donor can be considered, as results are generally comparable (although a recent study suggests an HLA-matched sibling donor may be preferred, when available) [19, 66]. No recommendation has been made by the ELN on the best myeloablative conditioning regimen [44]. Autologous HCT is not recommended by the ELN for patients without a suitable donor who are receiving intensive chemotherapy [44]; however, a recent study suggests cord blood HCT could be considered in MDS patients without a suitable donor [67]. Of note, a recent study reported that patients with MDS who had a hypomethylated epigenomic signature relative to their donor’s profile were more likely to relapse after HCT [68].

The optimal timing for HCT has not been defined, but it is generally accepted that blast percentage >10% before HCT is associated with a higher risk of relapse; thus, if bone marrow blasts are >10%, cytoreductive treatment prior to HCT is recommended (i.e., HMA or intensive chemotherapy) [43]. A study examining HCT strategies in MDS found the life expectancy of patients with low- or INT-1-risk MDS was higher when HCT was delayed somewhat but performed prior to transformation to AML. In contrast, higher life expectancy for patients with INT-2- or high-risk disease was achieved when HCT was performed as soon as possible after diagnosis [69].

The successful management of relapse following HCT depends at least in part on pre-HCT strategies, and the use of conventional cytoreductive chemotherapy prior to HCT to reduce the risk of relapse is still debated due to the potential to select for resistant clones. A recent study reported similar post-HCT outcomes in patients who proceeded directly to HCT and those who had received induction therapy with HMAs or conventional intensive chemotherapy [70]; however, these results need to be confirmed in additional studies.

#### Limitations of current therapies

Challenges relating to the treatment of MDS include chromosomal and/or molecular abnormalities that can cause pathophysiologic changes that influence the course of the disease but also offer a variety of therapeutic targets [12]. The number of approved drugs for MDS is limited, and not all agents have been shown to be highly efficacious and to improve survival [12]. Based on data from an MDS registry of 2377 patients, it was estimated that less than half (~44%) of patients with different MDS types are candidates for approved treatments [12]. Although many current approaches delay disease progression, they are not curative, and thus patients will require further treatment [12]. Finally, the same molecular aberrations that lead to “moving targets” in MDS may also eventually guide treatment; however, it is unclear how to incorporate molecular aberrations into the present treatment algorithm since there are few specific genotype-directed options available for MDS [40].

For patients with higher-risk MDS, initial responses to HMA therapy are limited (40–50%) and often short-lived [41]. Preliminary evidence from an analysis of the Surveillance, Epidemiology, and End Results Program (SEER)-Medicare database has estimated a median survival after HMA initiation of 18.4 months for patients with IPSS INT-1 disease and 11.6 months for patients with higher-risk disease, with transformation from high-risk MDS to AML after 19.3 months [71]. After HMA failure, median survival is approximately 5 to 6 months in the absence of HCT or novel clinical trials [43]. Further, real-world data for patients with high-risk MDS from the Spanish Cooperative Group on Myelodysplastic Syndromes (GESMD) registry between 2000 and 2013 suggest no notable improvement in survival during this period despite widespread use of azacitidine [72]. These observations indicate an urgent unmet need to improve outcomes with HMA-based therapy as well as an unmet need for patients with MDS after failure of first-line therapy, including management of ESA or HMA failure. One potential approach for the former is to identify factors predictive of response to HMAs [41]. Clinical markers have provided some insight, indicating that patients with peripheral blasts, poor performance status, high transfusion burden, and poor-risk cytogenetics have worse survival [73]. Studies examining molecular markers have provided mixed results [74–76], suggesting more research is needed [41].

#### Key therapies/regimens in development for MDS treatment

##### New approaches to treat low-risk MDS

There are multiple novel approaches currently being examined in patients with low-risk MDS. Most of these agents aim to manage anemia. The mechanisms of action and key results from clinical trials published to date are summarized in Table 2.

**Table 2:** Emerging therapies in MDS.

Agent/regimen	Phase	MoA	Patient population	Key results
**Low-risk MDS**				
Luspatercept [48]	3	Activin receptor fusion protein	IPSS-R very low-, low-, or intermediate-risk MDS-RS	Transfusion independence ≥8 weeks: 38% (vs. 13% with placebo; *P* < 0.001)
Sotatercept [47]	2	Activin receptor fusion protein	IPSS low- or INT-1-risk MDS	Hematologic response (erythroid): 49%
Roxadustat [77]	3	HIF prolyl hydroxylase inhibitor	IPSS-R very low-, low-, or intermediate-risk MDS and low RBC transfusion burden	Transfusion independence ≥56 days: 38%
Imetelstat [78]	2	Telomerase inhibitor	IPSS low- or INT-1-risk, RBC transfusion-dependent, non-del(5q) MDS that is relapsed/refractory to ESAs and HMA/lenalidomide naïve	Transfusion independence ≥8 weeks: 42%; Transfusion independence ≥24 weeks: 29%
CC-486 [83]	3	Oral azacitidine	IPSS low-risk, RBC transfusion-dependent MDS	Transfusion dependence ≥56 days: 31% (vs. 11% with placebo; *P* = 0.0002)
**High-risk MDS**				
Guadecitabine [86]	2	Second-generation HMA (decitabine linked to guanosine)	IPSS INT-2- or high-risk MDS after azacitidine failure	Hematologic response: 14.3%; primary azacitidine failure, low to high IPSS-R, and higher demethylation in blood were associated with better OS
ASTX727 [87]	3	Second-generation HMA (fixed decitabine and cedazuridine)	MDS by FAB classification; IPSS INT-1, INT-2, or high-risk MDS	Response rate: 61%; Complete response rate: 21%
Pevonedistat ± azacitidine [88]	2	NEDD8 inhibitor	IPSS-R high-risk MDS	Response rate: 79% (vs. 57% with azacitidine alone) Complete response rate: 52% (vs. 27% with azacitidine alone)
Enasidenib ± azacitidine [89]	2	IDH2 inhibitor	HMA-naïve and IPSS INT-2- or high-risk MDS; HMA relapsed/refractory	Response rate: 67%; 100% in HMA-naïve patients (enasidenib + azacitidine), 50% in HMA-failure patients (enasidenib alone)
Enasidenib [98]	1/2	IDH2 inhibitor	RAEB-1, RAEB-2, or IPSS-R high-risk MDS	Response rate: 53%; 46% in patients with prior HMA Complete response rate: 0%
Olutasidenib ± azacitidine [91]	1/2	IDH1 inhibitor	*IDH1*-mutated AML or IPSS-R intermediate-, high-, or very high-risk MDS	Response rate: 59%; 33% with olutasidenib alone, 73% with olutasidenib + azacitidine
Venetoclax ± azacitidine [92]	1b	BCL2 inhibitor	HMA relapsed/refractory MDS	Response rate: 7% with venetoclax alone; 50% with venetoclax + azacitidine
Venetoclax + azacitidine [100]	1b	BCL2 inhibitor	Treatment-naïve high-risk (IPSS score ≥1.5) MDS	Response rate: 70%
Durvalumab + azacitidine [92]	2	PD-L1 inhibitor	Treatment-naïve IPSS-R intermediate-, high-, or very high-risk MDS	Response rate: 62% (vs. 48% with azacitidine alone)
Atezolizumab ± azacitidine [93]	1b	PD-L1 inhibitor	Treatment-naïve or HMA-failure, IPSS-R intermediate-, high-, or very high-risk MDS	Response rate: 0% with atezolizumab monotherapy (HMA failure); 9% with atezolizumab + azacitidine (HMA failure); 62% with atezolizumab + azacitidine (HMA naïve)
Nivolumab or ipilimumab ± azacitidine [94]	2	PD-1/CTLA-4 inhibitor	Treatment-naïve or HMA-failure MDS	Response rate: 75% with nivolumab + azacitidine (HMA naïve); 71% with ipilimumab + azacitidine (HMA naïve); 13% with nivolumab monotherapy (HMA failure); 35% with ipilimumab monotherapy (HMA failure)
Sabatolimab + azacitidine or decitabine [95]	1b	TIM-3-targeted antibody	HMA-naïve IPSS-R high- or very high-risk MDS	Response rate: 63%, including 50% in high-risk MDS and 85% in very high-risk MDS
Rigosertib + azacitidine [96]	2	Multikinase inhibitor	Treatment-naïve IPSS INT-1-, INT-2-, or high-risk MDS	Response rate: 90%
Glasdegib + cytarabine/daunorubicin [99]	2	Hedgehog pathway inhibitor	Treatment-naïve AML or RA with excess blasts high-risk MDS	Complete response rate: 46% (includes patients with AML)
***TP53*-mutated MDS**				
Eprenetapopt + azacitidine [117]	2	Small-molecule inhibitor of apoptosis in *TP53*-mutated cancer cells	HMA-naïve, IPSS-R intermediate-, high-, or very high-risk *TP53*-mutated MDS and AML	Response rate: 75%
Eprenetapopt + azacitidine [111]	1b/2	Small-molecule inhibitor of apoptosis in *TP53*-mutated cancer cells	*TP53*-mutated MDS or AML with 20% to 30% bone marrow blasts	Response rate: 71%
Magrolimab + azacitidine [114]	1b	CD47-targeted antibody	Treatment-naïve, IPSS-R intermediate- to very high-risk MDS	Response rate: 100% (including 2 patients with *TP53*-mutated MDS)

*AML* acute myeloid leukemia, *BCL2* B-cell lymphoma 2, *CTLA*-4 cytotoxic T-lymphocyte antigen 4, *ESA* erythropoiesis-stimulating agent, *HIF* hypoxia-inducible factor, *HMA* hypomethylating agent, *IDH1* isocitrate dehydrogenase 1, *IDH2* isocitrate dehydrogenase 2, *INT-1* intermediate-1, *INT-2* intermediate-2, *IPSS* International Prognostic Scoring System, *IPSS-R* revised version of the International Prognostic Scoring System, *MDS* myelodysplastic syndromes, *MDS-RS* myelodysplastic syndromes with ring sideroblasts, *MoA* mechanism of action, *NEDD8* neural precursor cell expressed, developmentally downregulated 8, *OS* overall survival, *PD-1* programmed death-1, *PD-L1* programmed death-ligand 1, *RA* refractory anemia, *RAEB* refractory anemia with excess blasts, *RBC* red blood cell.

Roxadustat is a hypoxia-inducible factor prolyl hydroxylase inhibitor that is being investigated in a phase 3 study of anemia in patients with low-risk MDS and low RBC transfusion burden (NCT03263091 [77]) and a phase 2/3 study in patients with low-risk MDS (transfusion independent and ESA naïve; NCT03303066). Preliminary results of the phase 3 study showed that 9 (38%) patients with low-risk MDS who received roxadustat achieved transfusion independence for ≥56 consecutive days within the first 28 weeks (primary endpoint), with no major safety signals [77].

Imetelstat, a telomerase inhibitor, is currently being evaluated in a phase 2/3 study in RBC transfusion-dependent and ESA-relapsed or refractory low-risk MDS patients without the del(5q) phenotype, with encouraging results for 57 patients treated in the phase 2 portion. In the overall population, 8- and 24-week RBC transfusion independence rates were 37% and 23%, respectively, with a median duration of 65 weeks. For the subgroup of patients who were HMA and lenalidomide naïve, the 8- and 24-week RBC transfusion independence rates were 42% and 29%, respectively, with a median duration of 86 weeks. The most common adverse events for both the subgroup and overall populations were cytopenias, which were typically reversible within 4 weeks [78].

Thrombopoietin-receptor agonists, such as romiplostim or eltrombopag, are not formally approved for patients with MDS but may be a treatment option for thrombocytopenia in patients with blasts <5% [41]. Results of randomized trials in low-risk MDS patients treated with eltrombopag or romiplostim showed platelet responses in ~35–50% of patients, depending on the response criteria used [79, 80]. Although thrombopoietin-receptor agonists seem to be well tolerated, there are reports suggesting the risk of transformation to leukemia [81, 82]. In a randomized phase 2 study in patients with advanced MDS, AML transformation occurred in 31/50 (62%) eltrombopag-treated patients [81]. In a study in 60 patients with lower-risk MDS treated with romiplostim, the annualized rate of progression to AML was 2% [82].

A randomized phase 3 trial of CC-486, an oral azacitidine, vs. placebo in lower-risk MDS patients recently demonstrated 56-day RBC transfusion independence rates of 31% vs. 11%, respectively, with median durations of 11.1 vs. 5.0 months. CC-486 was also associated with more durable platelet and hemoglobin improvements, but also a higher frequency of hematologic toxicity and early deaths (primarily due to infection), which led to a decision to close further enrollment into the study [83]. ASTX727, a second-generation HMA consisting of a fixed-dose combination of cedazuridine and decitabine, is also being evaluated in phase 1/2 studies in lower-risk MDS (NCT03502668, NCT03906695).

While some patients with low-risk MDS respond well to immunosuppressive therapy with ATG or lenalidomide, more recent efforts are focused on modulation of the innate immune system and pro-inflammatory phenotype. The expression of TLRs is upregulated in the bone marrow of MDS patients and TLR signaling by MDSCs is integral to the pro-inflammatory microenvironment in MDS, and there has thus been some interest in employing therapies that target TLR signaling [30]. In addition, MDSCs express high levels of CD33, and therapies directed against CD33 to deplete MDSC counts in the bone marrow are in development, including both a monoclonal antibody against CD33 (BI 836858) that has been evaluated in preclinical models [84] and a novel bispecific tetravalent antibody against CD33 and CD3 (AMV564) that is being evaluated in a phase 1 study in patients with intermediate- or high-risk MDS after HMA failure (NCT03516591 [85]). Other approaches include the use of anti-inflammatory drugs, such as canakinumab (NCT04239157), which is currently being explored in combination with azacitidine in subgroups of low- and intermediate-risk MDS patients.

##### New approaches to treat high-risk MDS

Due to the high failure rate of HMA therapy among patients with high-risk MDS [71], there is an unmet need for additional options for patients failing HMA therapy. Multiple investigational therapies and regimens are being examined in this patient population (Table 2).

Second-generation HMAs are in clinical development for high-risk MDS. Guadecitabine has shown benefit in selected patients in a phase 2 study [86] and is being evaluated in a phase 3 study in patients with MDS failing first-line HMAs (NCT02907359). In the phase 2 study, among patients with higher-risk MDS or low-blast count AML after azacitidine failure, response was achieved by eight (14%) patients, including two patients with complete response, with an overall median duration of response of 11.5 months. Forty-four patients experienced serious adverse events, the majority (88%) of which were myelosuppression events [86]. ASTX727 (fixed-dose cedazuridine and decitabine) was recently approved by the FDA for the treatment of adults with MDS with various French-American-British subtypes and IPSS INT-1, INT-2, or high subtypes based on the results of a phase 3 trial in patients with MDS or AML who are candidates for intravenous decitabine (NCT03306264 [87]), which is ongoing. HMAs are also being evaluated with novel combination partners in both HMA-naïve and HMA-failure high-risk MDS patients, including pevonedistat (NCT03268954, NCT02610777 [88]), isocitrate dehydrogenase inhibitors (enasidenib [NCT03383575 ([89]), NCT03744390], ivosidenib [NCT02074839 [90], NCT03503409], and olutasidenib [FT-2102; NCT02719574 [91]]), venetoclax (NCT03404193), immune checkpoint inhibitors (durvalumab [NCT02775903 [92]], atezolizumab [NCT02508870 [93]], ipilimumab [NCT02530463 [94], NCT02890329], nivolumab [NCT02530463 [94]], and pembrolizumab [NCT03094637]), the anti-TIM-3 antibody sabatolimab (NCT03066648 [95]), and the multikinase inhibitor rigosertib (NCT01926587 [96], additional phase 3 study [97]); many of these HMA-based combinations have shown promising results (Table 2).

Early results from phase 1 and 2 studies are also showing encouraging efficacy with isocitrate dehydrogenase inhibitors (ivosidenib and enasidenib) as monotherapy in patients with *IDH1/2*-mutated high-risk MDS [89, 90, 98]. For example, among 17 patients with newly diagnosed or relapsed/refractory RAEB-1, RAEB-2, or IPSS-R high-risk MDS who had an *IDH2* mutation (NCT01915498), the overall response rate was 53%, with a median duration of response of 9.2 months [98].

Glasdegib, a Hedgehog pathway inhibitor, showed clinical activity in combination with cytarabine/daunorubicin (7 + 3) in a phase 2 study in patients with untreated AML or high-risk MDS, with a complete response rate of 46% [99]. A phase 2 study of glasdegib plus azacitidine in patients with untreated MDS, AML, and chronic myelomonocytic leukemia is ongoing (NCT02367456).

In early phase studies, the combination of venetoclax and azacitidine has shown benefit in patients with higher-risk MDS. In a phase 1b study (NCT02942290) in patients with treatment-naïve higher-risk (IPSS score ≥1.5) MDS, venetoclax plus azacitidine demonstrated a response rate of 70% [100]. In another phase 1b study (NCT02966782) in patients with relapsed/refractory MDS, a response rate of 50% and 7% was observed in patients in the venetoclax plus azacitidine and venetoclax alone arms, respectively [92].

CPX-351, a dual-drug liposomal encapsulation of daunorubicin and cytarabine in a synergistic 1:5 ratio, is approved for the treatment of patients with newly diagnosed therapy-related AML or AML with myelodysplasia-related changes [101], which includes patients with *de novo* AML who have an MDS karyotype. A subgroup analysis of the pivotal phase 3 study that supported its approval was performed in patients with oligoblastic secondary AML, often defined as bone marrow blasts 20–29%, which shares many biologic and clinical features with MDS [102]. For this subgroup of patients, CPX-351 improved median overall survival vs. 7 + 3 (12.50 vs. 5.95 months), supporting further exploration of CPX-351 in related disease groups, including higher-risk MDS. Treating MDS in patients who failed or are intolerant to initial HMA treatment with CPX-351 may overcome HMA resistance and sensitize the MDS cells to this treatment. This hypothesis is currently being tested in several studies of CPX-351 in MDS after HMA failure (NCT03957876, NCT04109690, NCT02019069, NCT03896269, NCT03672539). CPX-351 is also being evaluated as a first-line therapy in high-risk MDS (NCT03572764, NCT04061239) and in oligoblastic AML/MDS or MDS with excess blasts (NCT03393611, NCT04061239).

In addition to the HMA combination study mentioned above, rigosertib, a multikinase inhibitor, is being examined as monotherapy in a phase 3 study in patients with high-risk MDS and early HMA failure (≤9 months; NCT02562443), after results from a previous phase 3 study showed that this patient subgroup benefited most from rigosertib therapy [103]. However, the pivotal phase 3 study assessing the efficacy and safety of rigosertib in patients with high-risk MDS after failing prior HMAs did not meet its primary endpoint of improved survival [104].

FLT3 inhibitors, such as midostaurin and gilteritinib, are approved for patients with newly diagnosed or relapsed/refractory AML with an *FLT3* mutation, respectively, and may have therapeutic potential in MDS (NCT04097470, NCT04027309, NCT04140487) [105]. Notably, *FLT3* mutations are rarely seen in MDS and are frequently associated with the transformation of MDS to AML [106, 107].

The oral spliceosome modulator H3B-8800 has been tested in a phase 1 study that included patients with higher-risk MDS who were intolerant to or who had relapsed after HMAs (NCT02841540 [108]). Although H3B-8800 had a manageable safety profile, no complete or partial responses were observed [108].

Flotetuzumab, a CD3/CD123 antibody, has shown encouraging preliminary results in a phase 1 study in relapsed/refractory AML or INT-2- or high-risk MDS (NCT02152956), with an overall response rate of 43% [109].

Finally, bemcentinib is a highly selective inhibitor of the AXL receptor tyrosine kinase that is being investigated in a phase 2 study in patients with higher-risk MDS or AML who failed or were refractory to first-line HMA treatment (NCT03824080). Preliminary results from this study showed bemcentinib was well tolerated in patients with MDS and had a response rate of 33% [110].

*TP53* has been identified as a relatively common mutation in MDS patients [16] and is known to confer an adverse prognosis [19, 21, 22]. As a result, investigational strategies are targeting patients with *TP53-*mutated MDS. For example, eprenetapopt (APR-246) is a small-molecule inhibitor of apoptosis in *TP53*-mutated cancer cells that has been granted orphan drug designation for MDS by the FDA and the European Commission. In a phase 1b/2 trial, the combination of eprenetapopt and azacitidine was well tolerated in patients with *TP53*-mutated MDS or AML with 20–30% bone marrow blasts; median overall survival was 10.8 months, and the overall response rate was 71% [111]. Based on promising phase 2 results, the combination is currently being investigated in a phase 3 trial compared with azacitidine alone in *TP53-*mutated MDS (NCT03745716). However, a recent report indicated this trial failed to meet its primary endpoint of complete response rate [112]. Based on preclinical results showing its ability to target the p53 pathway by inhibiting MDMX and MDM2, ALRN-6924 is currently being evaluated in a phase 1 study as monotherapy and in combination with cytarabine for relapsed/refractory AML or IPSS-R intermediate-, high-, or very high-risk MDS with wild-type *TP53* (NCT02909972 [113]). The CD47-targeted antibody magrolimab (5F9) has shown robust clinical activity in combination with azacitidine in an ongoing phase 1 study in patients with intermediate- to very high-risk MDS (NCT03248479 [114]), particularly in patients with *TP53* mutations; expansion cohorts are ongoing, and registration trials in MDS are being initiated.

It is also reasonable to consider that recent developments in elderly AML patients may transfer to high-risk MDS, as these 2 diseases are thought to represent a “biological continuum” [41]. New or ongoing studies conducted in elderly patients with AML may therefore be applicable to patients with high-risk MDS. CPX-351 has demonstrated response rates of 47.7% in older patients with AML, and 34.0% of patients proceeded to HCT [115]; as noted above, CPX-351 is currently being evaluated in patients with high-risk MDS. The combination of azacitidine or decitabine plus venetoclax led to a 68% response rate in elderly patients with AML, with 21/145 (14%) patients proceeding to HCT [116]. Eprenetapopt in combination with azacitidine has been examined in elderly patients with *TP53*-mutated MDS or AML, with a 75% response rate [117]. However, further clarity and guidance are needed regarding how to apply such treatments to MDS.

## Understanding the role of monitoring for measurable residual disease (MRD) in patients with MDS

With the advances of combination treatments that can achieve high response rates, MRD-guided approaches have become an attractive therapeutic strategy for high-risk MDS patients. Treatment at molecular relapse is, for instance, more effective than at hematologic relapse after allogeneic HCT [118]; therefore, early detection is important and can be achieved with regular MRD monitoring after HCT. Sensitive MRD monitoring techniques are able to determine disease clonal and subclonal architecture and can detect relapse as early as possible [119]. For the best accuracy and specificity, a combination of next-generation sequencing-based monitoring and multicolor flow cytometric monitoring may be favorable [119]. Although various mutations have been studied as prognostic factors in MDS, it is unclear whether these mutations can be used as markers of MRD [119]. Mutations in genes such as *TP53*, *TET2*, *DNMT3A*, *IDH2*, and *RAS* have been associated with worse outcomes in patients with MDS, but further research is needed to determine how these mutations apply to next-generation sequencing and the prognostic significance and clinical efficacy of measuring pre- or post-HCT MRD in MDS [119, 120].

A universal MRD marker for MDS is unlikely because of the genotypic and phenotypic heterogeneity of the disease [119]. Thus, a more effective strategy may be individualized MRD monitoring using a targeted next-generation sequencing panel. Rautenberg and colleagues investigated the value of Wilms’ tumor 1 (*WT1*) as an MRD marker using a standardized, ELN-certified assay in patients with AML and MDS after allogeneic HCT [121]. *WT1* expression levels were measurable by standardized assay and predicted imminent relapse with high sensitivity and specificity in most patients with AML and MDS independent of genotype. However, the results from this retrospective analysis need to be confirmed in a prospective study.

The RELAZA2 trial evaluated MRD-guided azacitidine therapy for the prevention or delay of hematologic relapse in patients with MDS or AML and measurable MRD after first-line chemotherapy or allogeneic HCT [122]. Results from this study showed that MRD monitoring is useful for identifying patients who are more likely to relapse and that MRD-guided treatment may prevent or delay relapse [122].

An ongoing observational cohort study is evaluating individual molecular MRD monitoring for MDS patients after allogeneic HCT to develop highly sensitive methods for early detection of relapse based on patients’ unique mutations (NCT02872662). Another observational cohort study is being performed to develop assays to determine the impact of the therapy that patients receive for the treatment of AML or MDS and to determine if these tests can help identify those patients who are at greater risk of disease relapse (NCT01311258).

## Monitoring for progression of MDS to AML

The progression of patients from MDS to AML is relatively common, and regular follow-up visits, including bone marrow evaluations with cytogenetic analysis, are necessary for all patients with MDS; frequency of follow-up depends on disease risk and choice of treatment [44]. The time frame and frequency of monitoring for progression to AML depends on various factors but largely relies on the risk classification, including the molecular profile, of each patient and their response to treatment; studies have demonstrated that this risk can change over time [123]. Patients who progress to AML after MDS are categorized as AML with myelodysplasia-related changes; these patients differ with regards to their response to standard induction chemotherapy compared to *de novo* disease [32, 124].

## Summary and final conclusions

There is a tremendous unmet need for new treatments for MDS; rates of relapse are high, and many patients are not eligible for existing approved therapies. Patients should therefore be offered clinical trial options across all disease stages [41]. While many studies are evaluating agents with different mechanisms of action, most are still in early stages of development. The diverse nature of the genetic mutations that drive MDS and other myeloid disorders, ranging from clonal hematopoiesis of indeterminate potential to AML, means that therapies need to be developed for specific patient subsets. It is unlikely that there will ever be a universally effective treatment for MDS. As more is learned about the molecular pathophysiology of MDS, it is expected that more effective, personalized treatment options will become available.
